# Supplementary material for: Super-resolution microscopy reveals a Rab6a-dependent trafficking hub for rhodopsin at the mammalian rod photoreceptor Golgi
Source: Biol Open. 2025 Nov 25;14(11):bio062303. doi: 10.1242/bio.062303 (PMC12690528; doi:10.1242/bio.062303)
Supplement: Supplementary information [file biolopen-14-062303-s1.pdf]

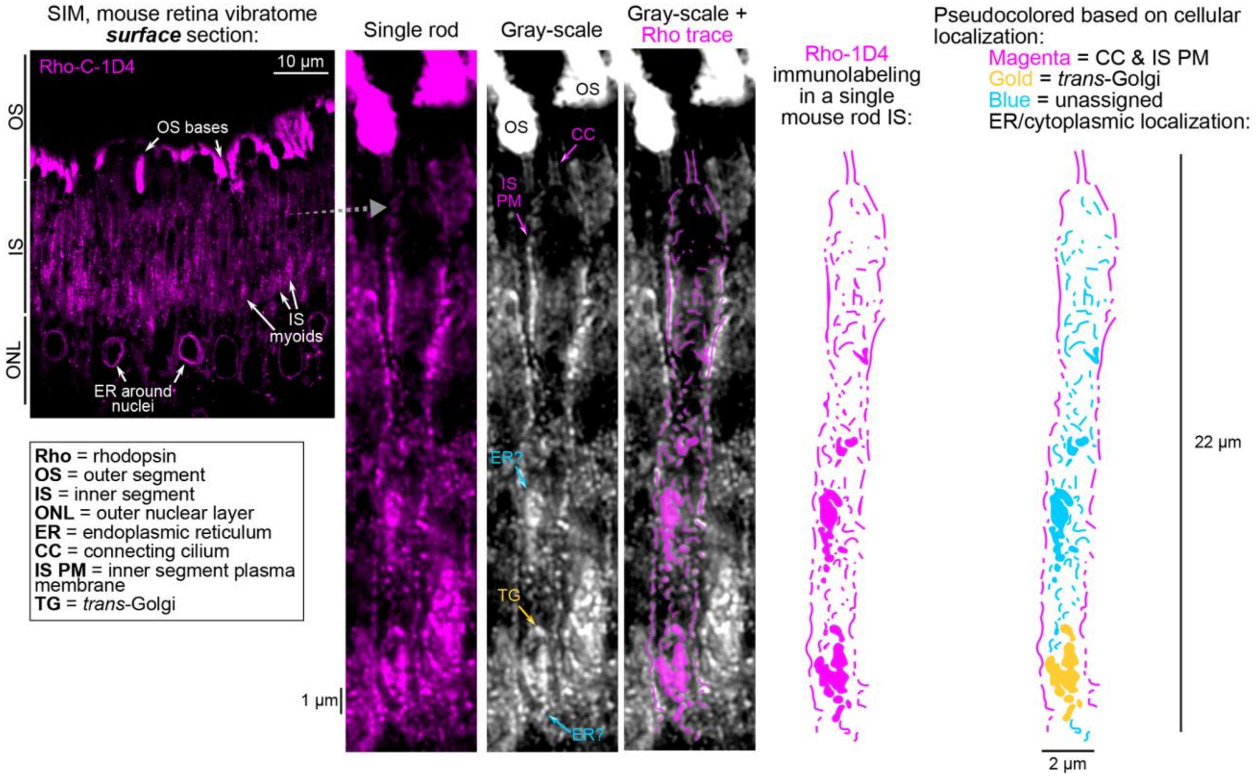

Fig. S1. Rhodopsin localization in a single mouse rod inner segment.

SIM Z-projection image of a WT mouse retina vibratome surface section immunolabeled for Rho-C-1D4 (magenta). In this region, Rho labeling was enriched in the IS myoid as indicated but was localized at the OS bases and in the ER around the nuclei of the ONL. The magnified view of an entire rod IS demonstrates the full Rho-C-1D4 labeling pattern, including an enrichment at trans-Golgi (TG), at the inner segment plasma membrane (IS PM), at the connecting cilium (CC), along with unassigned ER/cytoplasmic localized Rho. A legend is provided for all abbreviations used in the figure. SIM, structured illumination microscopy.

Biology Open • Supplementary information

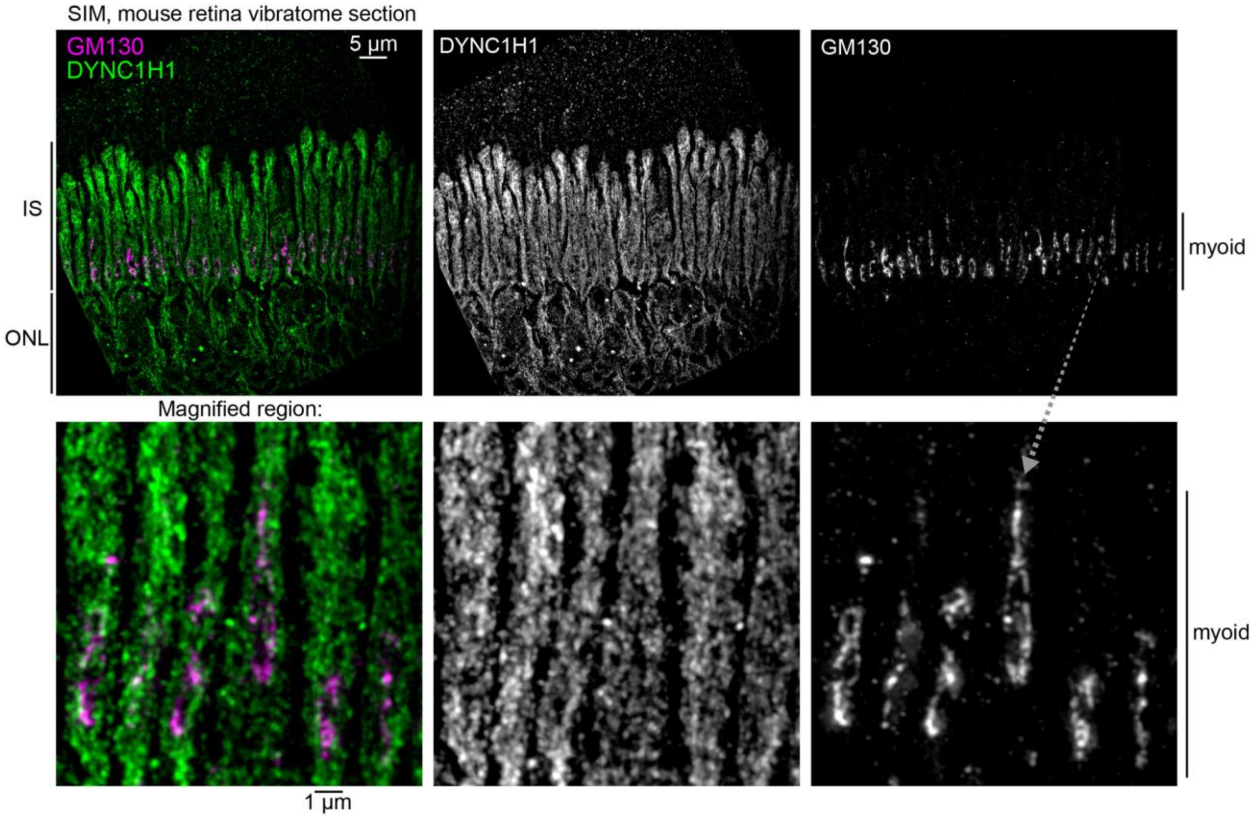

Fig. S2. DYNC1H1 SIM localization in WT mouse rods.

Shown is a SIM z-projection image of a WT mouse retina section that was immunolabeled for the dynein-1 complex heavy chain, DYNC1H1 (green), and GM130 (magenta). DYNC1H1 is localized throughout the IS and ONL; however, in the magnified panels, DYNC1H1 partially overlapped with the GM130 cis-Golgi. The magnified region is indicated with a dotted gray arrow. IS, inner segment; ONL, outer nuclear layer; WT, wild type; SIM, structured illumination microscopy.

Biology Open • Supplementary information

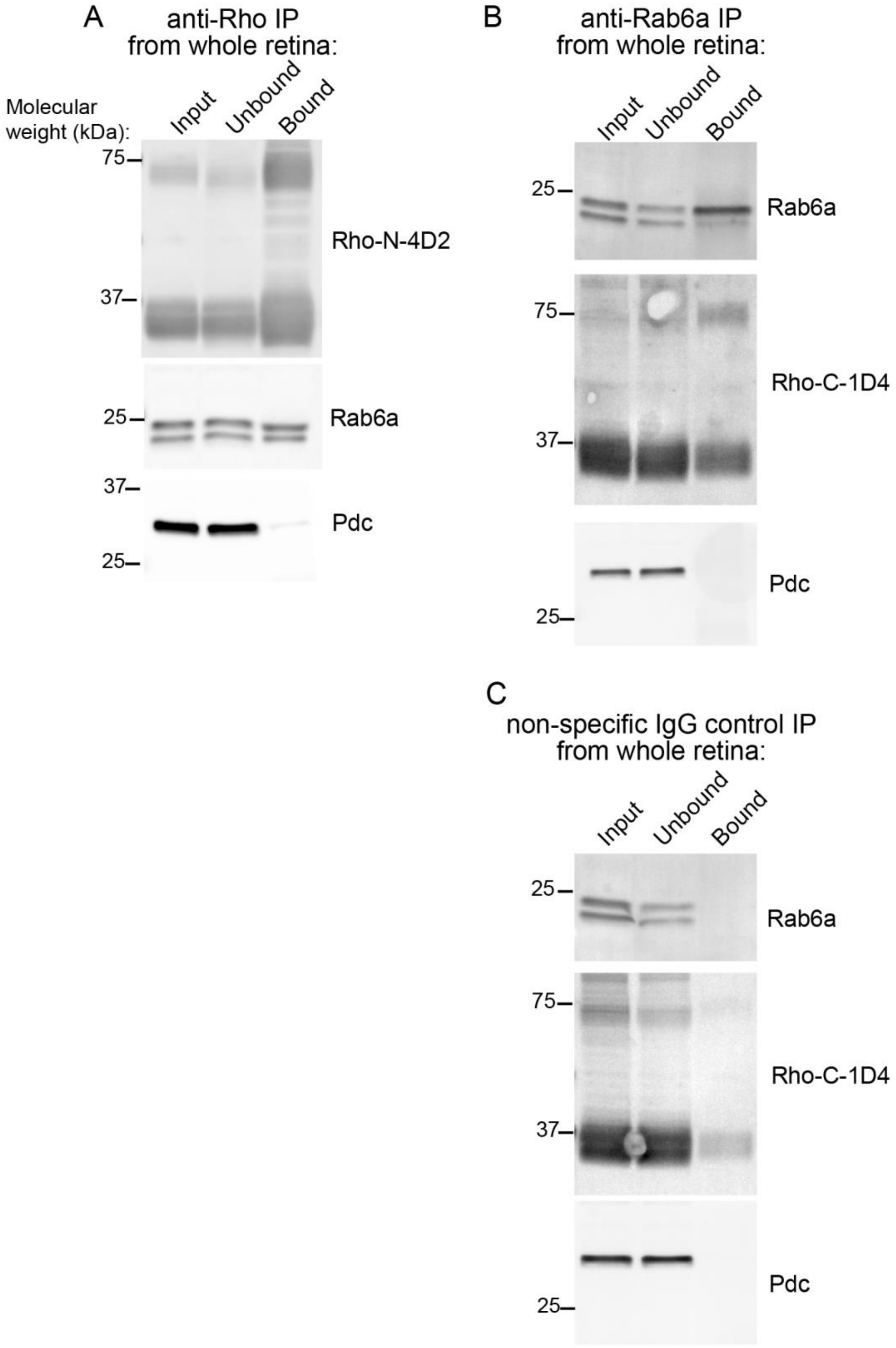
A
anti-Rho IP
from whole retina:
Molecular
weight (kDa):
Input
Unbound
Bound
75
37
Rho-N-4D2
25
Rab6a
37
Pdc
25
B
anti-Rab6a IP
from whole retina:
Input
Unbound
Bound
25
Rab6a
75
Rho-C-1D4
37
Pdc
25
C
non-specific IgG control IP
from whole retina:
Input
Unbound
Bound
25
Rab6a
75
Rho-C-1D4
37
Pdc
25

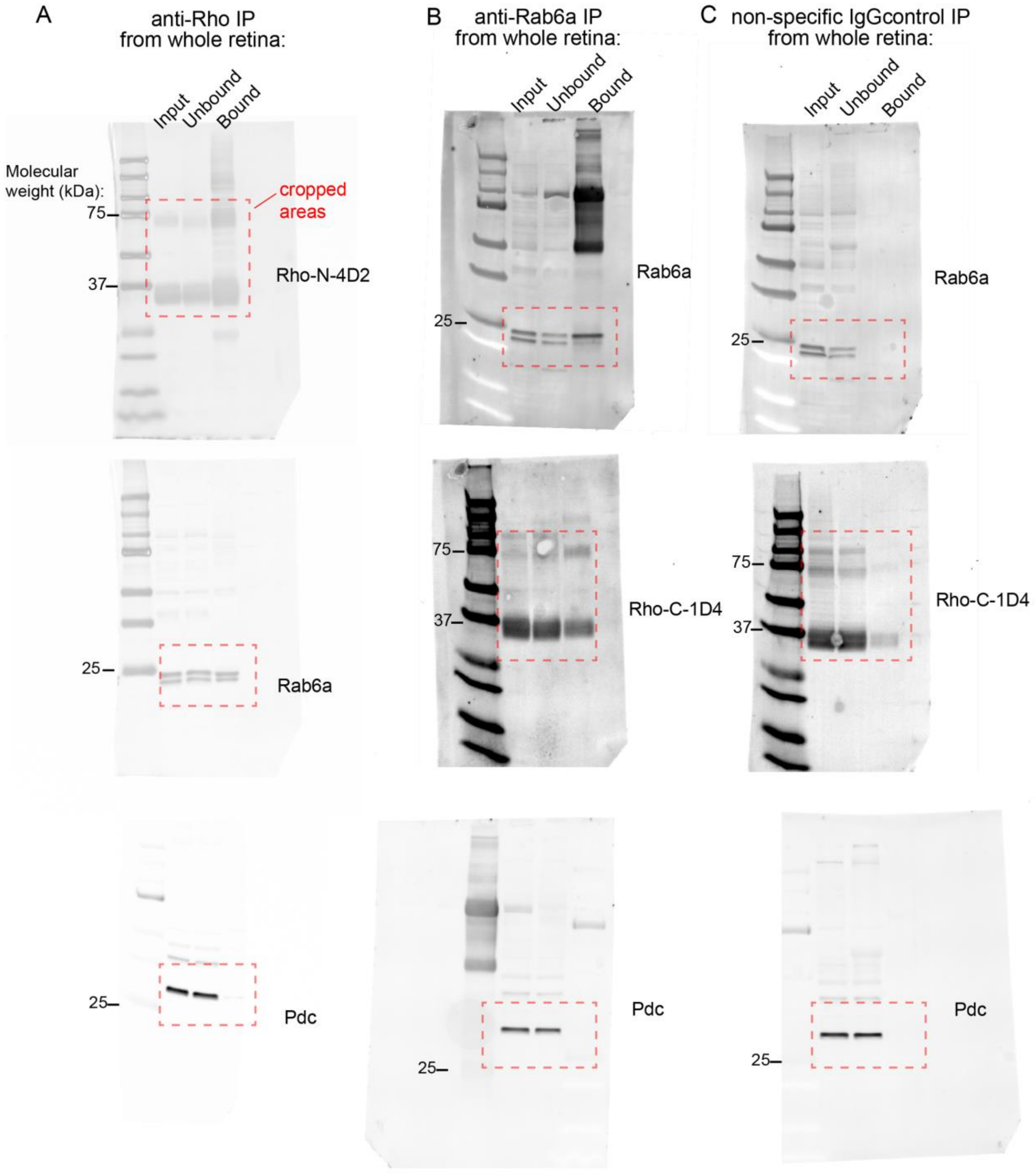

Fig. S3. Rab6a + rhodopsin co-immunoprecipitation (co-IP).

Co-IP results from WT mouse retinal lysates incubated with (**A**) anti-1D4 agarose beads ("anti-Rho IP"), (**B**) pre-bound Rab6a-IgG protein A/G beads ("anti-Rab6a IP"), and (**C**) non-specific IgG-bound control beads. In all western blots, input lanes correspond to 2.5% (% vol/vol) of the total starting lysate volume, unbound lanes correspond to 2.5% (% vol/vol) of lysate volume post

Biology Open • Supplementary information

bead incubation, and bound lanes correspond to half the total eluate from each co-IP bead sample. In Rho-N-4D2 and Rho-C-1D4 blots, the larger, ~70 kDa band is the dimer band, while the smaller, ~35 kDa bands represent monomeric Rho. Molecular weight marker sizes, in kilodaltons (kDa), are indicated to the left of each blot image. Antibodies used for western blot detection are listed to the right of each corresponding blot. IP, immunoprecipitation.

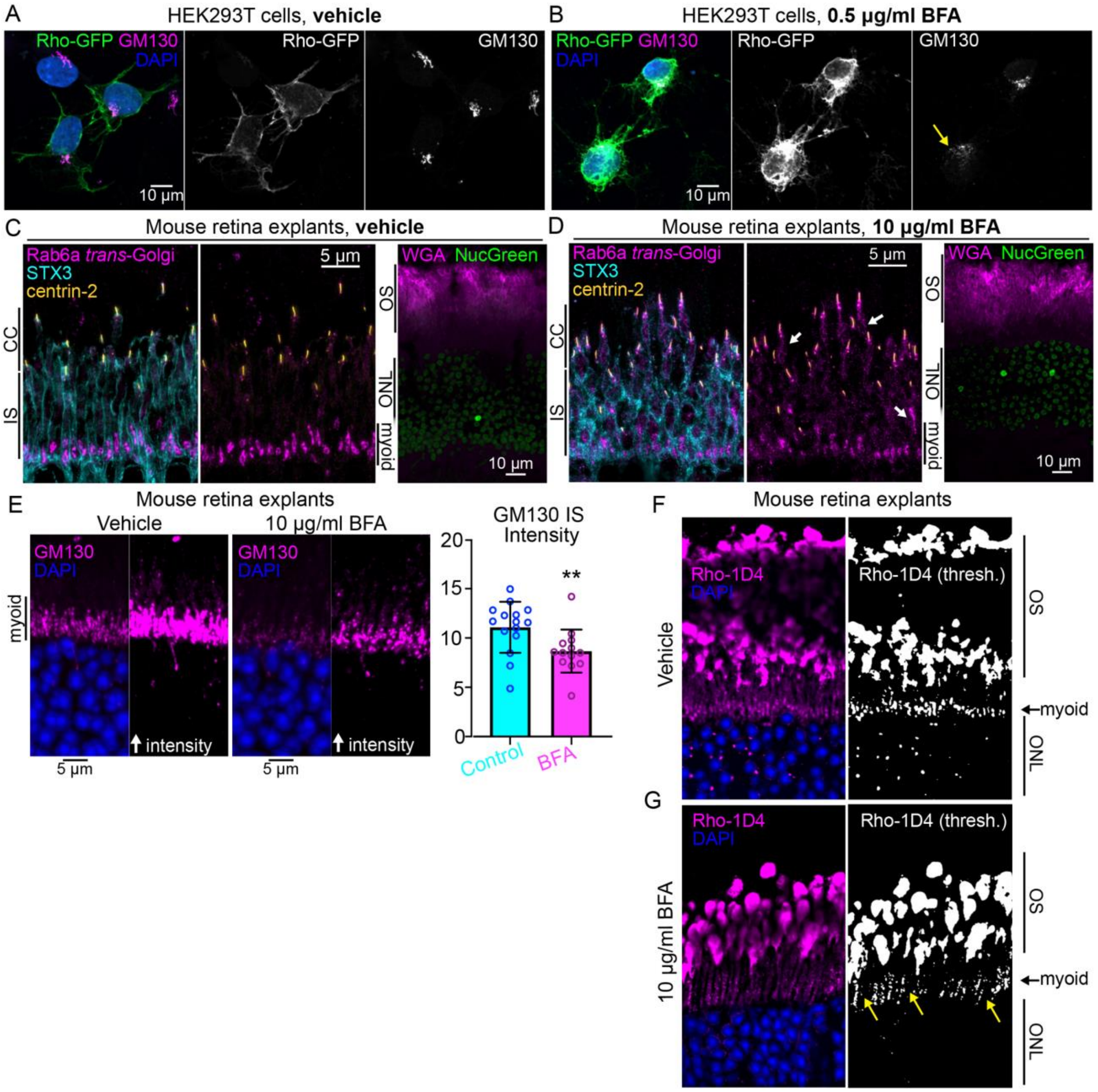

Fig.S4. Brefeldin-A has a minor effect on rhodopsin localization in mouse retina explants.

(**A, B**) BFA blocks post-Golgi Rho delivery to the plasma membrane in HEK293T. Confocal images of HEK293T cells transfected with Rho-GFP plasmid and treated with vehicle (DMSO, **A**) or (**B**) 0.5 µg/mL of BFA for 4 h at room temperature (RT). The cells were immunolabeled for GM130 (magenta) and DAPI (blue). In untreated cells, Rho-GFP (green) is predominantly localized at the plasma membrane. BFA caused Golgi disruption (B, right panel, yellow arrow)

Biology Open • Supplementary information

and Rho-GFP mislocalization. (**C,D**) Images of mouse retina explants treated with vehicle (**C**, DMSO) or 10 μg/ml of BFA (**D**) in oxygenated Ames' media for 2 h at RT. After fixation, cells were immunolabeled for Rab6a (cyan), STX3 (magenta), and centrin-2 (yellow). Rab6a (magenta) was properly localized to the IS myoids in untreated mouse retinal explants but was mislocalized throughout the IS, including near the connecting cilia, in BFA-treated mouse explants (D, middle panel, white arrows). STX3 labeling (cyan) was used to mark the IS plasma membrane. The right panels of both (**C**) and (**D**) show ex vivo retinal explants that were immunolabeled with NucGreen dye (green) post-treatment to demonstrate limited cell toxicity after either vehicle or BFA treatment. WGA (magenta) was used to mark OS. (**E**) Confocal images of mouse retina explants treated with either vehicle or 10 μg/ml of BFA and stained for GM130 (magenta) and DAPI (blue). BFA leads to Golgi fragmentation and a significant reduction in GM130 IS intensity (right panel graph, $**P<0.01$, unpaired t-test). Points on the graph = intensities from different IS regions (2 explant replicates were analyzed per condition), the graph's Y-axis = mean GM130 IS intensities (arbitrary units), bars = mean values, and error bars = standard deviations. (**F, G**) Confocal images of vehicle (**F**) and BFA-treated (**G**) retinal explants stained for Rho-C-1D4 (magenta). BFA-treated retinas have slightly disrupted Rho IS distributions (yellow arrows). IS, inner segment; ONL, outer nuclear layer; OS, outer segment layer; SIM, structured illumination microscopy.

Biology Open • Supplementary information
